# HIV knowledge among male labor migrants in China

**DOI:** 10.1186/s12889-015-1653-1

**Published:** 2015-04-02

**Authors:** Bo Yang, Zheng Wu, Christoph M Schimmele, Shuzhuo Li

**Affiliations:** International Business School, Shaanxi Normal University, Xi’an, Shaanxi Province 710049 P. R. China; Department of Sociology, University of Victoria, Victoria, BC V8W 3P5 Canada; Institute for Population and Development Studies, School of Public Policy and Administration, Xi’an Jiaotong University, Xi’an, Shaanxi Province 710049 P. R. China

**Keywords:** HIV knowledge, Labor migrants, China

## Abstract

**Background:**

This study described knowledge about HIV prevention and transmission among labor migrants in China and assessed the factors that associate with HIV knowledge.

**Methods:**

The study is based on primary data collected in Xi’an city, China. The study includes 939 male rural-to-urban migrants aged 28 and older. The multivariate analysis used OLS regression techniques to examine the correlates of HIV knowledge.

**Results:**

Most migrants know what AIDS/HIV is, but many have deficient knowledge about self-protection and the transmission routes of HIV. About 40% of migrants fail to understand that condoms decrease the risk of HIV infection. Higher levels of education and internet usage associate with better HIV knowledge. Migrants who have engaged in sex with commercial sex workers have better HIV knowledge than migrants who have never paid for sex. This includes better knowledge of self-protection.

**Conclusion:**

Labor migrants are a high risk population for HIV infection. Their lack of HIV knowledge is a serious concern because they are a vulnerable group for infection and their sexual behaviors are spreading HIV to other members of the population and across geographic areas.

## Background

From 1985–1988, the incidence of HIV in China was sporadic. Just 22 cases of HIV infection were identified in seven provinces, and each of these originated outside China [1]. Since then, the disease has reached all 31 provinces and is being spread from high-risk groups (e.g., migrants, sex workers, injection drug users) to other members of the population [1-3]. In the past, injection drug use and tainted blood products were the main sources of HIV infection, but sexual transmission accounts for the majority (85%) of new infections [4,5]. UNAIDS estimates that there are currently 780,000 cases of HIV in China, but other studies place this figure closer to 1.5 million [6,7]. Although China has a low prevalence of HIV (0.04–0.07%), it nevertheless is facing a potential HIV crisis [1,2]. Given China’s massive population, even though the proportion of people with HIV is comparatively low, the absolute number of individuals at risk of becoming infected with HIV threatens to eclipse the number of cases in high HIV-prevalence, sub-Saharan African countries [8].

China appears to be the next frontier of the HIV epidemic because it has millions of people vulnerable to infection via high-risk behaviors [3,4]. Most previous studies and interventions have focused on commercial sex workers and intravenous drug users, but the risk behaviors of China’s migrant or “floating” population is also a major concern [4,6,9-11]. China’s economic boom is stimulating large-scale rural-to-urban migration [4,12]. The number of internal migrants exceeds 260 million and the size of this population is expected to continue to grow because of rural–urban disparities in employment opportunities [4,13]. There is some evidence to suggest that the large size and risky behaviors of this population could contribute to a substantial increase in the national prevalence of HIV [14]. Most (70%) cases of HIV are observed among rural residents and are concentrated among males. The prevalence of HIV is about 1.8 times higher among rural-to-urban migrants than the stationary rural population [4].

Prior research demonstrates that the risk of contracting HIV among migrants is much higher than the national average and that migrants are contributing to the geographic spread of HIV [8,11,14,15]. A larger proportion of migrants than non-migrants report having multiple sexual partners and engaging in sex with commercial sex workers [8-10]. Several Chinese studies suggest that many migrants use condoms infrequently [2,8,9,14]. Moreover, the primary reason they give for condom use is contraception, with few using them for disease prevention [8,9]. Demographic attributes predispose migrants to risk behaviors, since migrants are predominantly young, unmarried males with limited formal education [14]. Most migrants are from rural areas where premarital sex was taboo and sexual behavior is highly regulated [8]. In urban areas, migrants are exposed to more permissive sexual norms and are relatively isolated from social control over their sexual behaviors. In addition, legal barriers and discrimination restricts migrants’ access to essential goods and services in host communities, which further increases their vulnerability [4].

The infrequent use of condoms for disease prevention suggests that migrants have a limited grasp of self-protection, in addition to facing other barriers to condom use, such as poverty and a lack of access to reproductive health services. Their risk behavior also appears to associate with a low perception of vulnerability to HIV infection [8,9,15]. Migrants represent a difficult to reach population for preventative interventions because of their mobility and limited access to health care services [4]. Poor knowledge of HIV/AIDS is a major determinant of exposure to infection as well as transmission to others [16]. Despite changing sexual behaviors, migrants lack knowledge about sexually transmitted infectious and safer sex practices [17]. This stems in part from poor sex education in schools as well as social norms that discourage discussion about sex and reproductive health [18]. The present study has two objectives. First, it examines knowledge about HIV among male labor migrants in China. The study describes their knowledge on the transmission and prevention of HIV and presents multivariate analysis of the correlates of their HIV knowledge. Second, the study compares migrants based on prior engagement with commercial sex workers to determine whether this risk behavior corresponds to differences in HIV knowledge.

## Methods

### Study sample

This study is based on primary survey data that our research team collected on labor migrants in Xi’an, China. Xi’an is the capital of Shaanxi province, located in north-central China. Xi’an is an emerging megalopolis and it attracts large volumes of labor migrants [19]. The survey was approved by the Research Review Board at the Institute of Population Studies, Xi’an Jiaotong University. The local government also approved this survey. At the time of the survey, the registered population of Xi’an was 8.5 million people and the city also hosted over 2 million migrants [20]. Our survey was conducted from December 2009 to January 2010. The target population of the survey was male rural-to-urban migrants aged 28 years and older. Our definition of migrants refers to people who left their registered place of residence (hukou) to reside and work in Xi’an.

The survey used a convenience sample comprised strictly of labor migrants. Migrants are dispersed throughout Xi’an and no local household registration information is available on them. This ruled out random and door-to-door sampling techniques. We recruited our respondents from three job banks (places where migrants go to find employment) and two construction sites. Local residents (non-migrants) at these job banks and construction sites were excluded from the survey. Before the interviews, researchers explained to the respondents that the data collected would be confidential and used strictly for academic purposes. The respondents were also informed of their right to withdraw from the interview. A self-administered questionnaire using the CAPI (computer-assisted personal interviewing) method was used. Because of the sensitive nature of the questions, respondents were provided with a private space to complete the interview. A total of 979 questionnaires were completed. The survey has a response rate of 97%, with 26 respondents withdrawing. Another 14 respondents (non-migrants) were removed for failing to meet the key selection criteria. The final study sample includes 939 respondents.

### Measures

The dependent variable is HIV knowledge. This included a screening question that indicated if the respondent had ever heard of HIV/AIDS. Respondents who gave a negative response were excluded from the follow-up questions. The follow-up included commonly used questions for measuring HIV knowledge [20]. These questions included two items on the prevention of HIV, seven items on the transmission of HIV, and two items about HIV misconceptions. A scale of 0–12 was created based on an affirmative answer to the screening question and correct answers to the follow-up questions. Respondents who reported never having heard of HIV/AIDS received a HIV knowledge score of 0. Those who heard of HIV/AIDS received a score of 1 and an additional 1 point for each correct answer to the 11 follow-up questions. A higher score on this scale indicates greater HIV knowledge. The Cronbach’s alpha for the scale is 0.80, which suggests good internal consistency.

The study includes several socio-demographic covariates. Table 1 presents the definition and descriptive statistics for the selected covariates. Age is measured in years. Marital status is measured as a categorical variable that includes never married, married, cohabiting, and divorced or widowed. Living arrangement is measured using a dummy variable that indicates whether the respondent lives with a spouse/partner or alone. Neighborhood is a categorical measure of the composition of the residential areas where migrants live: mostly non-migrant areas, areas where migrants are concentrated, areas mixed with migrants and locals (mostly non-migrants), and other areas. Education is measured in three levels: elementary school or less, junior high, and high school or higher. Religion is measured using a dummy variable that indicates whether the respondent has a religious affiliation.

**Table 1:** **Variable definitions and descriptive statistics of variables used in the analysis**

**Variables**	**Definition**	**M or %**	**SD**
Sociodemographic variables			
Age	In years (Range: 28–65)	39.1	7.29
Marital status	Never-married	13.4%	−
	Married	68.9%	−
	Cohabiting	8.4%	−
	Divorced or widowed	9.3%	−
Living arrangement	Dummy variable (1 = living with spouse or cohabiting partner, 0 = living alone)	34.2%	−
Neighborhood	Mostly non-migrants	23.1%	−
	Areas where migrant workers are concentrated	20.9%	−
	Residential areas mixed with local residents and migrants	50.1%	−
	Others	5.9%	−
Education	Elementary school or less	18.1%	−
	Junior high	58.6%	−
	High school or higher	23.3%	
Religion	Dummy variable (1 = have any religious affiliation, 0 = none)	27.5%	−
Migration history			
Place of origin	Dummy variable (1 = Shaanxi province, 0 = otherwise)	79.3%	−
Age at leaving home to work	In years (Range: 8–64)	22.14	7.55
Lifetime sexual experience			
Ever had sex	Dummy variable (1 = yes, 0 = no)	85.7%	−
Ever had commercial sex	Dummy variable (1 = yes, 0 = no)	19.4%	−
Number of sexual partners			−
0	Dummy variable (1 = yes, 0 = no)	14.3%	
1	Dummy variable (1 = yes, 0 = no)	45.1%	−
2	Dummy variable (1 = yes, 0 = no)	15.1%	−
3	Dummy variable (1 = yes, 0 = no)	8.8%	−
4	Dummy variable (1 = yes, 0 = no)	3.5%	−
5	Dummy variable (1 = yes, 0 = no)	2.9%	−
6–9	Dummy variable (1 = yes, 0 = no)	3.6%	−
10 or more	Dummy variable (1 = yes, 0 = no)	6.6%	
Self-reported health			
Good	Dummy variable (1 = yes, 0 = no)	34.7%	−
About average	Dummy variable (1 = yes, 0 = no)	47.6%	−
Poor	Reference group	17.7%	−
Ever used internet	Dummy variable (1 = yes, 0 = no)	45.1%	−
*N*			939

Note: Weighted means or percentages, unweighted N.

Source: The Xi’an Study of Reproductive Health among Male Migrant Workers.

The study considers two aspects of migration history. Place of origin is a dummy variable that indicates whether the respondent came from inside or outside Shaanxi province. Most migrants (79%) are from Shaanxi. The study also considers age at migration. The mean age of migration is 22 years. Three measures of lifetime sexual experience are used: ever had sex, ever had commercial (paid for) sex, and number of sexual partners (ranging from 0 to 10 or more). Table 1 shows that 86% of respondents have had sex and 19% have had commercial sex. About 45% of migrants have had one sexual partner and about 40% have had multiple sexual partners. Self-reported health is measured as a categorical variable: good, average, or poor. A dummy variable is used to measure if the respondent ever used the internet.

### Statistical procedure

Since the dependent variable (HIV knowledge) is a continuous variable, we used OLS models for the data analysis. To examine model adequacy, we assessed various OLS assumptions (e.g., collinearity, nonlinearity, normality, outliers, and heteroskedasticity) and experimented with alternative modeling techniques (e.g., robust regression). We found no evidence for violation of these assumptions.

## Results

### Bivariate results

Table 2 presents the responses to the questions on HIV knowledge among migrants. The table presents the percentage of correct responses for the full sample as well for a subdivided sample of those who have and have not engaged in sex with a commercial sex worker (CSW). Slightly more respondents who have had sex with a CSW (94%) reported that they have heard about HIV/AIDS than those who have not (91%), but this intergroup difference is statistically non-significant. Table 2 demonstrates that migrants have fairly low HIV knowledge. The mean HIV knowledge score (on a 12-point scale) for all migrants is 4.92. There is a significant difference between those who have had sex with a CSW and those who have not (*p* < 0.001). Those who have had sex with a CSW have comparatively more knowledge about HIV. The mean scores on the HIV knowledge scale are 5.73 for those with who have had sex with a CSW and 4.73 for those who have not.

**Table 2:** **HIV knowledge among male migrant workers: Xi’an, China, 2009–2010**

**All**		**Migrants with commercial sex experience**	**Migrants without commercial sex experience**	***t*** **-test on difference by experience of commercial sex (p-value)**
**HIV knowledge items**	**% of giving correct answers**	**% of giving correct answers**	**% of giving correct answers**	
Ever heard of HIV/AIDS (1 = yes, 0 = no)	91.8	94.51	91.15	0.138
Prevention				
Risk will be decreased when having sex with only one partner without HIV (T)	33.76	44.77	31.01	0.003
Risk will be decreased by condom use (T)	60.44	73.84	57.1	0.000
Transmission				
Handshaking with HIV positive people can transmit (F)	61.25	68.6	59.42	0.083
Eating with HIV positive people can transmit (F)	45.01	47.09	44.49	0.332
Blood donation in a hospital or donation vehicle will spread HIV (F)	44.90	45.35	44.78	0.985
Sharing table wares with infected people can transmit (F)	39.68	47.67	37.68	0.028
Swimming with HIV positive people can transmit (F)	30.97	31.98	30.72	0.820
Mosquito bite will spread HIV (F)	20.65	24.42	19.71	0.224
HIV positive women still can have healthy babies (T)	13.92	18.02	12.9	0.082
Misconceptions about HIV				
A man looks healthy may have HIV (T)	52.09	58.72	50.43	0.143
HIV can be healed (F)	33.76	45.35	30.87	0.002
Sum of HIV knowledge items except for perception of HIV risk.				
(range from 0–12)	4.92	5.73	4.73	0.000
*N*	939	182	757	

Note: Weighted means or percentages, unweighted N. T = the statement is true; F = the statement is false.

Source: The Xi’an Study of Reproductive Health among Male Migrant Workers.

Under half (45%) of labor migrants who have had sex with a CSW understand that having a single sexual partner (who is HIV negative) reduces the risk of HIV infection. This compares to under one-third (31%) of migrants who have never had sex with a CSW. There is also a significant intra-group difference in knowledge about condom use. Among migrants who have had sex with a CSW, almost three-quarters understood that using condoms reduces the risk of HIV infection. In comparison, among those who have not had sex with a CSW, just 57% understood this. Table 2 also illustrates that labor migrants have limited knowledge about the transmission routes of HIV and a large proportion have misconceptions about HIV. For the most part, there is not much difference between migrants who have and who have not had sex with a CSW on these aspects of HIV knowledge.

### Multivariate results

Table 3 presents the multivariate OLS regression of HIV knowledge levels among all migrants. The divorced/widowed have less HIV knowledge than the married. There is a significant association between place of residence and HIV knowledge. Migrants living in areas mixed with local and migrants have a higher HIV knowledge compared to others. Those from Shaanxi province have lower HIV knowledge than those from outside the province. Education is also an important risk factor. Migrants with junior high and high school and above have higher HIV knowledge compared to those with elementary school or less. Respondents who have a religious affiliation have higher HIV knowledge than migrants without a religious affiliation. Migrants who have ever had sex have higher HIV knowledge compared to those who have never had sex. Finally, internet usage also correlates with higher HIV knowledge.

**Table 3:** **Ordinary least squares model of HIV knowledge among male migrant workers: Xi’an, China, 2009-2010**

**Predictors**	**b**	**95%**	**CI**
Sociodemographic variables			
Age	-0.008	-0.039	0.022
Marital status			
Never married	-0.320	-0.908	0.268
Cohabiting	-0.303	-0.974	0.368
Divorced or widowed	-0.640*	-1.276	-0.005
Married (reference)			
Living arrangement			
(1 = living with spouse or cohabiting partner)	-0.198	-0.584	0.189
Neighborhood			
Areas where migrant workers are concentrated	0.224	-0.310	0.757
Residential areas mixed with local residents and migrants	0.700**	0.251	1.149
Others	0.596	-0.213	1.405
Mostly non-migrants (reference)			
Education			
Junior high	1.121***	0.648	1.593
High school or higher	1.891***	1.334	2.448
Elementary school or less (reference)			
Religion (1 = have any religion)	0.487*	0.092	0.881
Migration history			
Place of origin (1 = Shaanxi province)	-0.594*	-1.033	-0.156
Age at leaving home to work	-0.003	-0.028	0.022
Sexual experience			
Ever had sex (1 = yes)	0.932**	0.374	1.491
Ever had commercial sex (1 = yes)	0.503	-0.034	1.041
Number of sexual partners	0.021	-0.052	0.095
Self-reported health			
Good	0.433	-0.085	0.951
About average	0.360	-0.132	0.851
Poor (reference)			
Ever use internet (1 = yes)	0.881***	0.464	1.299
Intercept	2.659*	1.086	4.231
R squared	0.140		
*N*	939		

***p < 0.001; **p < 0.01; *p < 0.05.

Note: Unstandardized adjusted regression coefficients.

Source: The Xi’an Study of Reproductive Health among Male Migrant Workers.

Table 4 re-runs the multivariate analysis, presenting separate analysis for migrants who have had sex with a CSW and those who have not. For migrants who have had sex with a CSW, education is an important risk factor. Those with junior high and high school or higher, have much higher levels of HIV knowledge compared to those with elementary school or less. Those from Shaanxi province have lower HIV knowledge than those from outside the province. Internet usage also correlates with higher HIV knowledge among migrants with who have had sex with a CSW.

**Table 4:** **Ordinary least squares models of HIV knowledge among male migrant workers by commercial sex experience: Xi’an, China, 2009-2010**

	**Migrants with commercial sex experience**			**Migrants without commercial sex experience**		
**Predictors**	**b**	**95%**	**CI**	**b**	**95%**	**CI**
Sociodemographic variables						
Age	0.023	-0.040	0.086	-0.017	-0.051	0.018
Marital status						
Never married	0.503	-0.596	1.601	-0.595	-1.300	0.110
Cohabiting	-0.097	-1.302	1.108	-0.333	-1.151	0.485
Divorced or widowed	-1.141	-2.484	0.202	-0.484	-1.212	0.244
Married (reference)						
Living arrangement	-0.099	-1.032	0.833	-0.229	-0.658	0.200
(1 = living with spouse or cohabiting partner)						
Neighborhood						
Areas where migrant workers are concentrated	0.634	-0.603	1.872	0.067	-0.534	0.669
Residential areas mixed with local residents and migrants	0.520	-0.554	1.593	0.692**	0.190	1.194
Others	-0.190	-1.911	1.531	0.835	-0.091	1.760
Mostly non-migrants (reference)						
Education						
Junior high	1.669**	0.531	2.807	0.950***	0.418	1.482
High school or higher	2.312***	1.037	3.588	1.788***	1.157	2.419
(reference: elementary school or less)						
Religion (1 = have any religion)	0.640	-0.271	1.552	0.476*	0.032	0.919
Migration history						
Place of origin (1 = Shaanxi province)	-1.157*	-2.121	-0.192	-0.413	-0.914	0.088
Age at leaving home to work	-0.033	-0.096	0.029	0.006	-0.022	0.033
Sexual experience						
Ever had sex (1 = yes)	-	-	-	0.901**	0.295	1.507
Number of sexual partners	0.031	-0.065	0.127	0.006	-0.114	0.126
Self-reported health						
Good	0.358	-0.838	1.554	0.384	-0.199	0.967
About average	-0.313	-1.482	0.857	0.454	-0.094	1.001
Poor (reference)						
Ever use internet (1 = yes)	1.063*	0.106	2.021	0.834**	0.364	1.304
Intercept	3.565*	0.054	7.077	2.866**	1.112	4.619
R squared	0.228			0.116		
*N*	181			756		

***p < 0.001; **p < 0.01; *p < 0.05.

Note: Unstandardized adjusted regression coefficients.

Source: The Xi’an Study of Reproductive Health among Male Migrant Workers.

The correlates of HIV knowledge for migrants who have never had sex with a CSW are somewhat different. Higher levels of education associate with better HIV knowledge. Internet usage also associates with increased HIV knowledge. Where these migrants differ from migrants who have had sex with a CSW is in the correlations between HIV knowledge and religious affiliation and neighborhood. These factors are non-significant for migrants with who have had sex with a CSW. For migrants who have not had sex with a CSW, living in areas mixed with locals and migrants associates with higher HIV knowledge compared to migrants living in other types of neighborhoods. Those with a religious affiliation have higher HIV knowledge than those without a religious affiliation.

## Discussion

The vulnerability of labor migrants to HIV infection is partially associated with their limited HIV knowledge. In China, there are institutional, sociocultural, and policy barriers that limit the dissemination of knowledge about reproductive health to migrants [4]. This study documented the level of HIV knowledge among male migrants in Xi’an city, China and examined the factors that contribute to HIV knowledge among them. Knowledge is a key factor in the prevention of HIV infection since it associates with self-protection behaviors as well as misconceptions about vulnerability to infection [4]. Unfortunately, our findings are not encouraging. While most (92%) migrants have heard of HIV/AIDS, the average level of knowledge of the prevention and transmission of HIV is deficient. Compared to rural non-migrants, labor migrants have less HIV knowledge, which partly stems from a lack of preventative interventions that target migrants, in addition to socioeconomic vulnerabilities (e.g., low education, poverty) among migrants [4,9,11,15]. Although our data was collected in 2009–2010, no policies have been implemented to substantially address these issues since then.

Migrants tend to engage in riskier sexual behaviors than non-migrants [8,11,14,15]. In our study population, over 40% of male labor migrants have had multiple sexual partners and over 19% have engaged in sex with commercial sex workers. The proportion of migrants reporting multiple sexual partners is somewhat lower than Anderson et al. and Li et al. reported for labor migrants in other cities (Beijing, Nanjing, and Shanghai), but higher than reported on migrants in other studies, which may reflect differences in study design [8,14,15]. The observed proportion in our sample is attributable to our focus on lifetime rates and males aged 28 years and older. The proportion of our sampled respondents who paid for sex is also higher than observed for male migrants in Beijing and Nanjing (10%) [10]. Our findings demonstrate that most migrants do not clearly understand the link between having multiple sexual partners and the risk of HIV infection. Moreover, 40% of migrants do not know that condom use decreases the risk of HIV, which is consistent with previous studies that demonstrate that condom usage is low and is seldom used for self-protection [2,8,9,14]. Anderson et al. report that only 14% of migrants stated that disease prevention was the purpose of using condoms [14].

Our results also demonstrate that educational attainment is a major determinant of HIV knowledge. Part of the reason migrants are vulnerable to HIV infection is the low educational attainment among this group. In our sample, just 23% of migrants have higher school or more education. The migrant population also lacks access to public health services [21]. These services are provided only to urban residents with local household registration status (hukou), which most migrants do not possess and have great difficulty obtaining. These factors contribute to different risk perceptions between migrants and non-migrants. What is notable from our findings is that internet usage and living in areas where migrants are not predominant associates with an increase in HIV knowledge. The correlation between internet usage and HIV knowledge implies that the internet could be serving as an informal source of education about AIDS/HIV and self-protection. Living in areas without high concentrations of migrants could improve HIV knowledge because these areas provide greater opportunities for migrants to access local family planning and prevention services.

Our study also compared HIV knowledge between migrants who have engaged in sex with commercial sex workers (CSW) and migrants who have not. Male clients of sex workers are a high risk group for HIV transmission [10]. Migrants are more likely than non-migrants to seek out commercial sex workers and this risk behavior partially accounts for the comparatively higher prevalence of HIV among them [11]. Our bivariate findings demonstrate that migrants who have had sex with a CSW have significantly higher HIV knowledge than migrants who have not had sex with a CSW. This intragroup difference in the total HIV knowledge score is primarily attributable to greater knowledge about self-protection, since the differences on the questions about HIV transmission routes and misconceptions are mostly small and statistically non-significant.

Almost three-quarters of migrants who have had sex with a CSW understand that using condoms decreases the risk of HIV transmission. This compares to 57% of migrants who have not had sex with a CSW. This finding is consistent with previous research that demonstrates that male clients of sex workers have a higher perception of vulnerability to HIV infection and other sexually transmitted diseases [11]. In our multivariate analysis, there is non-significant difference in HIV knowledge between migrants who have had sex with a CSW and those who have not. Our findings suggest that factors such as marital status, education, religion, and sexual experience account for the relatively higher HIV knowledge among migrants who have had sex with a CSW. Further research is needed to pinpoint the relative importance of these factors and investigate why the male clients of commercial sex workers have a higher perception of vulnerability.

The HIV knowledge of labor migrants should be situated within a broad context of vulnerability. Yang demonstrates that post-migration economic marginalization, social isolation, and lax social control combine to increase their vulnerability [22]. For labor migrants, economic hardship and exploitation are common experiences. Labor migrants who have low HIV knowledge and engage in risky behaviors tend to work long hours for little compensation [23]. Many labor migrants reside in sub-standard and crowded housing conditions, leading to the neglect of personal hygiene [4]. Migrants are also a socially isolated group, concentrated in neighborhoods and workplaces that consist mostly of other migrants, with few opportunities to interact with non-migrants. Hence, migrants are both cut-off from their home villages (social networks) and poorly assimilated into their host communities [22]. This separation from their families and disconnection from mainstream society can weaken social control over their behavior. Yang argues that frustration over blocked economic opportunities and social detachment increases the likelihood that labor migrants will engage in risky behaviors.

### Limitations

This study has several limitations. First, the variables used to assess sex risk behavior measured the lifetime rates of number of sexual partners and sex with a CSW. Some of these activities could have occurred prior to migration. This should not bias our regression estimates since our concern is how a migrant’s sexual history correlates with their HIV knowledge. We are unconcerned with the timing of their previous sexual encounters. However, the recall bias associated with lifetime measures of sexual behavior could lead to an under- or overestimation of the number of sexual partners [24]. Second, this study is limited by its reliance on a convenience sample. As noted above, labor migrants are a difficult to reach population, and it nearly impossible to randomly sample this group. Third, no data was collected on drug use, which is a well-established determinant of HIV infection. Finally, our study sample was restricted to migrants aged 28 and older at the time of the survey, and thus our findings are not generalizable to the younger migrant population.

## Conclusion

There are clear advantages to increasing HIV knowledge among labor migrants [20]. Although migrants are well-known to engage in high-risk sexual behaviors, few policies have been developed to address this problem. Given the large size and mobility of this population, interventions aimed at decreasing the HIV-related risk behaviors of migrants is a key strategy for China’s fight against the HIV epidemic. This is important for increasing their self-protection as well as protecting their sexual partners. One potential way to achieve this is to loosen the restrictions that tie household registration status to access to health care services. The term “floating population” is used to refer to labor migrants because these people are transients and are excluded from public services in their temporary places of residence. There are millions of migrants who have left their hometowns for work. Rural-to-urban migrants desperately require access to amenities, but face unequal treatment while working away from home.
